# The Influence of E-Learning and Emotional Intelligence on Psychological Intentions: Study of Stranded Pakistani Students

**DOI:** 10.3389/fpsyg.2021.715700

**Published:** 2021-08-25

**Authors:** Fahad Alam, Qing Yang, Muhammad Yaseen Bhutto, Nadeem Akhtar

**Affiliations:** ^1^School of Economics and Management, University of Science and Technology Beijing, Beijing, China; ^2^Business School, Shandong Jianzhu University, Jinan, China; ^3^School of Urban Culture, South China Normal University, Foshan, China

**Keywords:** e-learning, emotional intelligence, study stress, burnout, performance

## Abstract

The COVID-19 pandemic has forced the government to close the educational institutions globally, to contain the infection of the disease, which has affected the academic activities of local and international students significantly. This unexpected shift from offline classes to online learning has created psychological disruption among the students. At that backdrop, this study aims to investigate the influence of e-learning and emotional intelligence (EI) on the study stress, burnout, and performance of Pakistani students by applying emotion regulation theory. The data (*N* = 387) is based on international students, enrolled in 10 different universities in China. The results indicate that both e-learning and EI have significantly affected perceived study stress, burnout, and performance of students. These findings have provided evidence that online classes and EI can influence study stress, burnout, and performance of students. The study concludes that EI has significant impact on the psychological pressure of a student.

## Introduction

Psychological pressure is the foremost impediment to academic success. Psychological stress can impact the inspiration, concentration, awareness, and social interactions of students, which are considered critical factors for students to attain academic success (Unger, 1998). The COVID-19 pandemic crisis has brought into focus the psychological pressure of various affected populations. Due to the coronavirus, strict precautions and delays in starting colleges and universities worldwide are expected to affect the mental health and well-being of students (Cao et al., 2020; UNESCO Education, 2020). Emotions are considered an important trait of the psychology of a student. Students are bound with different emotionally demanding states, such as homework, assignments, quizzes, examinations, and deadlines, are connected that encompass their psychological intentions (Tyng et al., 2017). Students are expected to show patients and positive intentions toward the study. Positive emotions have enhanced the psychological behaviors of students that lead to better outcomes (Corradino and Fogarty, 2016). A few studies have reported the psychological impact of the COVID-19 epidemic on the general community, medical staff, patients, children, and adults (Chen et al., 2020; Yang et al., 2020). However, no comprehensive study on the psychological well-being of Pakistani students, facing the COVID-19 crises, has been conducted to date. Therefore, it is critical to explore what aspects can ease the high psychological pressure rate among students? Although much research has investigated the emotional influences on study devotion, learning and memory, and problem-solving in education sectors for long-term sustainability (Vuilleumier, 2005; Shen et al., 2009; Um et al., 2012), few are acknowledged the impact of e-learning (online classes), and emotional intelligence (EI) on psychological intentions of students, such as study stress, burnout, and performance (Berenson et al., 2008; Han and Johnson, 2012). The present study tends to expand on what is previously explored and identified the effect of e-learning and EI on the psychological pressure of students, such as study stress, burnout, and performance.

The feelings and emotions of students are directly linked with their study and career achievements, though career achievements demand a standard performance. Scholars realized that motivations of students direct their behavior toward educational achievements and academic success (Van Tilburg and Igou, 2013; Wigfield and Gladstone, 2019), but psychological pressure in terms of stress, tension, fear, and various psychosomatic problems are connected with a variety of destructive outcomes (Naquin and Gilbert, 1996). The psychological pressure is experienced when physical and emotional requirements of academic activities do not meet the competencies, needs, and resources of the students (Curran and Standage, 2017); hence, due to the COVID-19 outbreak, students educational activities have been dramatically affected in various aspects, such as online classes, postponing of physical events, and students mobility (Altbach and de Wit, 2020; Tesar, 2020), which caused strict travel restrictions and thousands of students psychological pressure influence their behavior negatively in term of canceling their plans for studying overseas. For instance, according to the Institute of International Education, nearly 90% of the US institutions have estimated a decrease in foreign student enrolment (Martel, 2020); British Council in 2020 indicated that 39% of Chinese scholars, which is a major source of overseas students in the United Kingdom, are hesitant about withdrawing their study plans (Durnin, 2020). Similarly, 59.95% of international students in China come from Asia, including more than 28,023 students from Pakistan (Ministry of Education in China, 2019). As a result, significant adverse psychological pressure has been reported among the study stress, burnout, and performance of students (Wang et al., 2020). No such research has been conducted in Pakistan yet. Therefore, this study aims to expand the existing literature by assessing the effect of e-learning (online classes) and EI on the psychological pressure (study stress, burnout, and performance) of students among overseas Pakistani students stranded in Pakistan.

The influence of online classes on work stress, burnout, and performance can be moderated by EI, but little existing research has observed direct and indirect impacts among these variables. The present study aimed to investigate the influence of e-learning (online classes) on work stress, burnout, and performance, and the potential moderation of EI of these effects. This research is based on emotion regulation theory (Gross, 1998), indicating that individuals assess the understanding, valence, and value relevance to control their feelings and emotions according to the existing situation. A person who possesses high emotional competencies is better tends to execute his or her skill (Kirk et al., 2008). A very valuable mechanism by which negative outcomes of sentiments can be coped is EI. Goleman (2001) recommended that individuals who possess strong social awareness are better at minimizing the destructive consequences of sentiments, and their overall performance also increased substantially. Garg et al. (2016) found that EI was directly related to adjustment to existing situations, and adjustment was directly connected to overall performance.

During uncertain situations, the EI supports individuals to identify their expectations and how to act appropriately. Students have to face such unanticipated situations quite often. They need to show positive feelings and emotions, such as patient, attention, hope, and prestige and encounter the requirement of their academic syllabus. These challenging situations enhance psychological pressure and might affect work performance, study stress, and burnout. However, EI can help to increase the work performance of students and simultaneously eliminate the effect of burnout and study stress.

## Conceptual Framework and Hypotheses Development

### Emotional Intelligence

The concept of EI was originated in the 1920s, but it came to attention when it was categorized properly by Salovey and Mayer (1990). They stated, “the capability to observe one's own and other's mental state and sentiments, distinguish among them, and practice this information to guide individuals' thinking and actions” (Krishnan et al., 2018). EI intends psychological comfort and accomplishment in life (Carmeli et al., 2009), such as academic success (Petrides et al., 2004), work performance (Koman and Wolff, 2008), and work-related stress (Mikolajczak et al., 2007). By examining the link between EI and psychological pressure, Stevens et al. (2019) found that emotionally intelligent individuals are more confident, happy, and sociable; consequently, their EI traits inversely affect academic stress among students.

By examining the link between EI with psychological pressure coping with styles, Erözkan (2013) found that a high level of EI significantly coping with psychological pressure stress among students. Similarly, Fteiha and Awwad (2020) highlighted the positive relationship between EI and stress coping with methods in University students. Their research indicated that individuals with higher EI possess a greater capacity to withstand psychological pressure. Despite much research on the impact of EI on different work outcomes, the influence of EI on student performance, burnout, and study stress has been marginally considered in Pakistan.

### Emotional Intelligence, Work Stress, Burnout, and Performance

Emotional intelligence is the internal capability of a human to understand manage the self-sensitive actions and reactions of an individual. Bar-On et al. (2006) refer to EI as a group of non-cognitive abilities and skills, which diminish the environmental demands and pressures. A number of scholars found that EI skills effectively handle pressure situations more successfully. The academic requirements are extremely demanding and stressful; therefore, it is practical to suggest that EI coping with the psychological pressure of students and enhance their performance (Austin et al., 2005; Por et al., 2011). Besides other environmental and emotional demands, students have to handle academic demands, such as exams, assignments, presentations, maintain grades, and motivation for an academic career. In addition, the study of Enns et al. (2018) revealed that high EI is connected with good pressure management. He suggested that an emotionally intelligent individual can handle psychological pressure situations with a more efficient way to encounter challenges at school. Mohzan et al. (2013) found that the characteristics of high EI are related to positive educational development. They claimed that EI is dynamic to the health and academic success of a student.

The academic career of a student is associated with high work stress and burnout (Yucha et al., 2009; Jenaabadi et al., 2017). In an emerging country like Pakistan, due to the lack of resources, poor working environment, energy crises, and poor management, stress levels and burnout of a student further aggravate (Ali, 2012). Consequently, the physical and emotional problems of a student negatively affect their learning capabilities and academic performance. Friedman (2014) argued that excessive psychological pressure might arise physical problems for a student and possibly diminish his/her intellectual abilities. Karimi et al. (2014) study point out that the overall response to fight against stress and burnout is deeply reliant on feelings and emotions. Students having a high level of EI can attenuate burnout and work stress (Cazan and Năstasă, 2015) increase satisfaction level (Runcan and Iovu, 2013) and professional development (Năstasă, 2010). EI increases the psychological pressure resilience, well-being, and academic performance of students (MacCann et al., 2020).

Although it has been more than 15 months since Chinese universities were closed due to the coronavirus pandemic, student physical absenteeism increased due to stranded in their home countries, which caused psychological pressure, poorer academic achievement, huge gaps in practical skills, and development (Coe et al., 2020). Accordingly, Bonal and González (2020) indicated that not going to universities decreases learning opportunities and accelerate stress level for all, but specifically students from low developed nations. Suhaimi et al. (2014) study revealed that EI plays a big role in controlling crises in an emergency. Fiorilli et al. (2020) have posited that EI strongly prevents stress and burnout of students, enhancing academic performance. Bar-On et al. (2006) found that higher than average intellectual skills are more successful in coping with environmental and psychological pressure situations. EI indicated a direct effect to reduce the burnout level among the students during stressful environments; consequently, a high level of EI was significantly correlated with the performance of students (Yusoff et al., 2021). It is reasonably acceptable that several studies explored the relationship between EI on the work outcomes of nurses (Ioannidou and Konstantikaki, 2008; Alonazi, 2020; Mo et al., 2020). However, this study investigates the influence of EI on the performance, study stress, and burnout of students while stranded in Pakistan due to the COVID-19 crises. Therefore authors hypothesized:

*H1: There is a significant positive relationship between emotional intelligence and work performance among students*.
*H2: There is a significant adverse relationship between emotional intelligence and study stress among students*.
*H3: There is a significant negative connotation between emotional intelligence and burnout among students*.

### Online Classes, Study Stress, Burnout, and Performance

The educational institution is advanced in the last few years, which is proved by the immense use during this COVID-19 (Chatterjee and Chakraborty, 2021). A lot of online platforms are available for classes (Nash, 2020). It was a challenge for an educational institution to organize its educational curriculum online. Mishra et al. (2020) stated that before the pandemic, online classes existed in advanced countries. However, no institution was prepared for a complete switch to online classes. Empirical research has proved that students feel that their learning skills improved through online classes as compared to physical education (Bojović et al., 2020), but the response is different from the perspective of Pakistani students. According to Williams et al. (2011), online learning systems are inadequate due to experiencing many challenges. Proper network adaptability is not possible, especially in rural areas where Internet facilities are barely found, and the learning experience is entirely different from physical classes (Williams et al., 2011). Adnan and Anwar (2020) observed the psychological burden of Pakistani students toward online courses during COVID-19. Their findings highlighted that online education can negatively affect desired performance in underdeveloped countries like Pakistan. Their psychological pressure influences the inability to access the Internet, technical issues, financial issues, and other educational resources, such as experimental labs (Adnan and Anwar, 2020).

The coronavirus pandemic is accompanied by strict measurement that has led students to confine in their homes; an alarming social life and education in quarantine have put students under psychological pressure. The lack of group learning activities, lab work activities, and experimental work is experienced by both instructors and students due to the online classes. All this has led to psychological stress and burnout by both students and teachers. Less physical involvement and spending most of the time at home create an immense negative impact on the performance of students (Chandra, 2020). Rohman et al. (2020) argued that online classes enhanced academic pressure, which directly impacts health, decision-making power, psychosomatic complaints, sleeping difficulties, worrying about the future, anxiety, depression, workload, etc. of students. According to Sahu (2020), the COVID-19 pandemics have brought many psychological shocks and a negative influence on the psychological well-being of students, which directly led to acute work stress and anxiety (Aktekin et al., 2001). Cao et al. (2020) examined the psychological influence on University students in China during the coronavirus pandemic. They found out a negative impact on the performance and a high level of psychological burdens for the students. The previous studies heightened that uncertainty negatively impacts the academic development of students and influences the psychological pressure of students (Bayram and Bilgel, 2008; Wang et al., 2020).

Jæger and Blaabæk (2020) argued that online classes discouraged the learning competencies of students due to discrimination compared with better family facilities. As universities adopted online classes (Yen, 2020), the query arises “how this approach benefits students with lower-income families and remote areas?” Where several students belong from lower-income families (Fry and Cilluffo, 2019). Due to a lack of facilities and experimental lab work, students from rural and lower-income families have limited or no access to online classes. Similarly, the financial cost is another obstacle to take online classes (Adam et al., 2020). Sundarasen et al. (2020) study highlighted the significant contributor to stress and burnout was the sudden shift to online classes. They documented the financial constraints, online classes barriers, academic performance, and uncertainty about the future due to the lockdowns negatively affected the psychological intentions of students. According to Choi (2020), millions of students are worried about academic loss due to the unproductive way of learning. Hence, online classes indicated a strong impact on the psychological pressure and performance of a student (Jiang et al., 2021). Therefore, we constructed the following hypothesis:

*H4: There is a significant negative relationship between online classes and work performance of student*.
*H5: There is a positive relationship between online classes and study stress*.
*H6: There is a positive association between online classes and student burnout*.

## Theoretical Framework

Emotional intelligence is considered a pillar for educational, psychological, and management studies. The theory is rooted to understand oneself emotions and emotional reactions and identifying the experiences of different feelings and sentiments (Bliss, 2006). It also refers to tackling emotional disappointments, adapting behaviors, and the ability to avoid emotional stress, burnout, and learning to evade the negativity of feelings and emotions (Chandra and Mathur, 2016, p. 231). Richards and Pryce (2006) propose that individuals with a high level of EI are more proficient in reducing stress levels, burning out, and improving their performance. Higher level of EI more appropriately coping with the stress and burnout sources, which enhance work performance (Alonazi, 2020). According to Sadovyy et al. (2021), individuals with a high level of EI precisely use their emotional skills and abilities to diminish stress and burnout related to the pandemic. Accordingly, the work performance of emotionally intelligent individuals is superior to those with a low level of EI during the current pandemic (Alonazi, 2020; Rezvani et al., 2020). Educational activities are now switched into the new environment to maintain the development of students (Paloş et al., 2010). During the last decade, emotion has been considered a significant aspect of the learning process; past studies show that EI can stimulate the academic performance of students (Cleveland-Innes and Campbell, 2012). There is evidence that the emotional competencies of a student are associated with online learning. Students with emotional skills are expected to react more effectively than students with a low EI level. The emotional competencies reduce student psychological pressure, and consequently, the learning performance of students can be improved (McKnight, 2013). Similarly, Enns et al. (2018) found that a high EI is linked with better stress management. According to Enns et al. (2018), an emotionally intelligent individual can cope with stressful situations and encounter educational challenges effectively.

Previous studies on emotion regulation indicated that EI is directly associated with psychological management across multiple professions, including student learning (Pugh, 2008; Ergur, 2009) and academic performance (Williford, 2010). EI capabilities facilitate students to adapt the uncertain situation accurately and encouraging them to identify innovative solutions, which might influence their intellectual skills that lead to standard academic performance. According to Berenson et al. (2008), EI strongly moderates academic performance in distance and online classes. Another study by Grandey (2000) found that high levels of EI diminish work stress, burnout, and increase performance levels. Similarly, Wu et al. (2007) study results concluded that EI shows an effective role in reducing psychological pressure, interpersonal and environmental conflicts, and increasing positive work behavior and outcomes.

**Figure 1:**
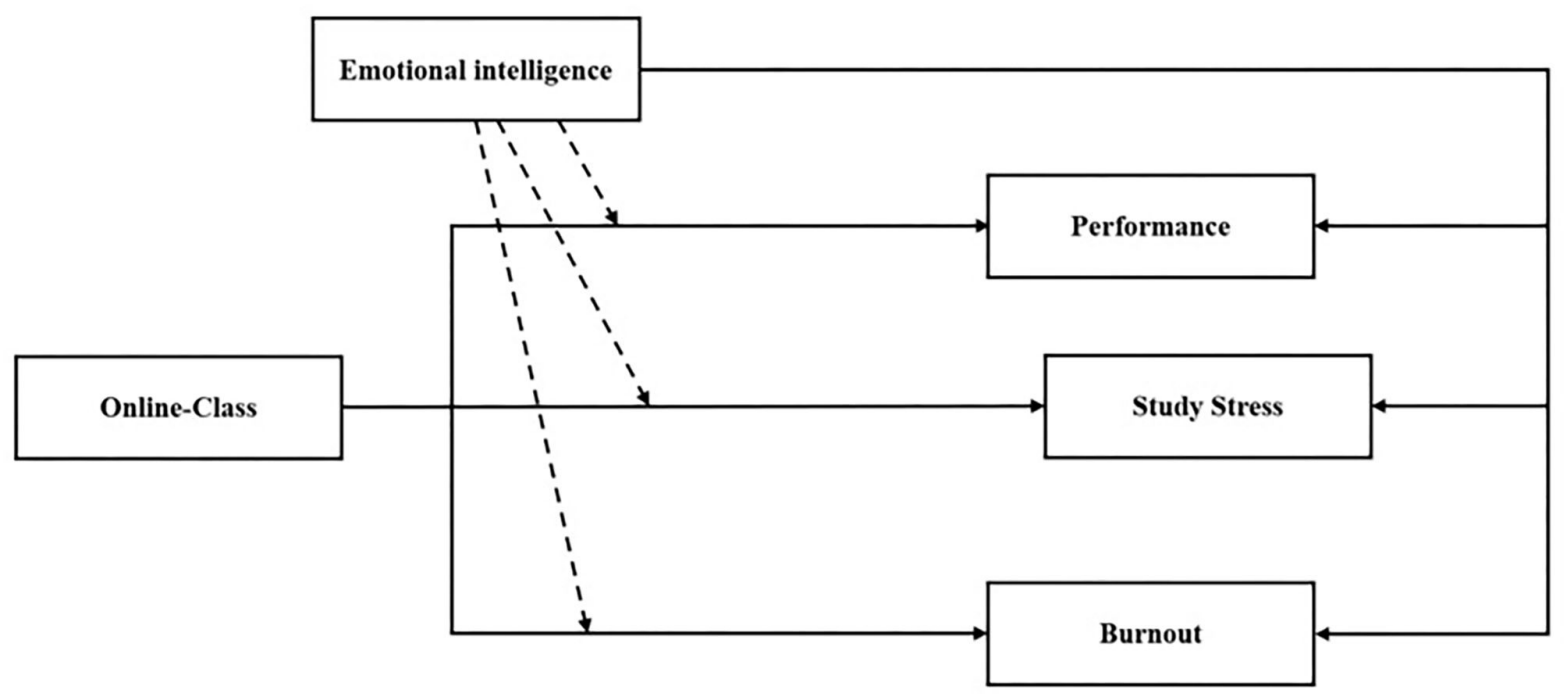
Theoretical framework.

An increase in EI is connected to a decrease in stress. In the same vein, Márquez et al. (2006) collected a sample of students in Spain and found that higher EI is significantly associated with academic achievement. As stated above, psychologists claim that individuals who have EI skills are more successful than those who do not have EI. Despite its contributions toward success in other areas, there have a few studies conducted of EI as a predictor for success in online classes. The online classes continue due to COVID-19, which plays a greater role in the academic curriculum of students. Likewise, higher EI is connected with better psychological functioning (Zeidner and Matthews, 2018; Zysberg and Raz, 2019). Therefore, the authors investigate the possible moderating role of EI between online classes, study stress, burnout, and performance, as shown in Figure 1.

*H7: Emotional intelligence moderates the impact of online classes on student's performance*.
*H8: Emotional intelligence moderates the impact of online classes on student's study stress*.
*H9: Emotional intelligence moderates the effect of emotional dissonance on student burnout*.

## Research Methods

### Data Collection Process

The study has used a quantitative approach to collect the data from stranded Pakistani students, due to COVID-19 and restriction of cross-border movements, studying at various universities in China. The survey was divided into two sections: (a) the construct measurement items and their corresponding measurement scales, and (b) demographic variables, as shown in Table 1. The convenience sampling technique was employed because it is appropriate when it is difficult to obtain a complete sampling frame. This type of sampling is suitable because it permits a theoretical generalization of the findings. To ensure valid data collection, the authors hired trained research assistants, who used different social platforms to collect the data, such as Google surveys and social media platforms like Facebook, WeChat, and WhatsApp. Before filling the questionnaire, participants were asked to give their voluntary consent to participate in this study. Out of 429 filled questionnaires, the authors found 387 questionnaires valid and useful for the present study.

**Table 1:** Demographic characteristics of respondents.

**Variables**		**Frequency(*N* = 387)**	**Percentage (%)**
Gender	Male	296	76.5
	Female	91	23.5
Age of Respondents	18–25	148	38.2
	26–30	164	42.4
	30–35	62	16
	36–40	13	3.4
Education (currently pursuing)	Undergraduate	102	26.4
	Master	189	48.8
	PhD	96	24.8
Geographical representation	Eastern	189	48.83
	Western	67	17.31
	Northern	83	21.5
	Northern	48	12.4
Marital status	Single	169	43.7
	Married	212	54.8
	Others	6	1.5

### Measurements

#### Emotional Intelligence

To measure EI, the Schutte Emotional Intelligence Scale was used, developed by Schutte et al. (1998). This test contained 32 self-report items. Students were asked to rate their agreement with all 32 statements using a 5-point Likert scale ranging from “1 = strongly disagree, 5 = strongly agree.” A sample item include “When I am in a positive mood, I motivate myself and solving problems is easy for me.” The Cronbach's alpha of the EI scale was 0.88.

#### Stress

The items for psychological stress were precisely developed to measure students' stress manifestations during the coronavirus pandemic. It includes seven items on a 5-point Likert scale rage, 0 “Not at all stressful” to 4 “Extremely stressful.” The designed instrument was constructed based on emotional and psychological models of stress (Lazarus and Folkman, 1984). Each question was developed to cover the specific stress domains that were subjected to the COVID-19 pandemic (i.e., study burden, social isolation, relationship with colleagues and professors, classroom studying, and on-campus physical activities). A sample item contains “I feel frustration due to too much work and lack of resources.” The Cronbach's alpha of this scale was 0.83.

#### Burnout

To measure burnout of students (Schaufeli et al., 2002), 15-item scales were used, which include three dimensions: exhaustion (five items), depersonalization (four items), and personal accomplishment (six items). A sample items include “Do you feel exhausted because of your online classes” and “how often do you feel emotionally drained during the classes.” The overall Cronbach's alpha of the burnout scale was 0.87.

#### Performance

To assess the academic performance of students, 12-item scales measuring well-being, emotionality, self-control, and social skills of a student were adopted from the study of Cooper and Petrides (2010). The questions were constructed based on the EI correlations with the academic scores (e.g., I truly regulate my emotions, which can facilitate me to perform efficiently). The Likert scale ranging from “high-performance score” (0) to “poor performance” (4). The Cronbach's alpha of this scale was 0.89.

#### E-Learning

To consider this measurement (Chakraborty et al., 2020), 11-item scale of e-learning was used to capture the academic performance, study stress, and burnout of a student (e.g., Learning in Physical classrooms are better than an online class). Moreover, students were enquired to specify how frequently they feel satisfaction or dissatisfaction. A Likert scale ranged from “frequently satisfaction” (0) to “frequently dissatisfaction” (4). The overall Cronbach's alpha was 0.86.

## Data Analysis

Before analyzing the study hypotheses, we tested confirmatory factor analyses (CFAs) to compute the discriminant validities of reports of e-learning, EI, study stress, burnout, and academic performance of students. To analyze the model fit of CFA, we must consider the standard criteria of the various model fit indices. It has been recommended that root means square error of approximation (RMSEA) values <0.05 are better, however, values between 0.05 and 0.08 are indicated a satisfactory level of model fit (Mulaik et al., 1989). The CFI value that is close to 0.90, the Normed fit index (NFI) value and the incremental fit index (IFI) value that exceeds 0.90 recommended a satisfactory model fit (Bentler, 1990; Byrne, 1994). The root means square residual (RMR) value range 0–1 is acceptable, but a value < 0.05 is considered well fit (Byrne, 1994). We run λ^2^ differences among a single-factor and five-factor model to check which model appropriately fit to the data sample. The estimated five-factor model provided [χ^2^ (113) = 268.4, *p* < 0.001, NFI = 0.914, CFI = 0.943, IFI = 0.952, RMR = 0.01, and RMSEA = 0.04] better results as compared with four-factor model shaped by linking study stress and burnout (χ^2^ (116) = 585.6, *p* < 0.05, NFI = 0.825, CFI = 0.847, IFI = 0.838, RMR = 0.04, and RMSEA = 0.08) and more better compared to a single-factor model (CFI = 0.517, NFI = 0.567, IFI = 0.518, and RMSEA = 0.12).

## Results

To measure the discriminant validity refers to the amount to which the measures do not reflect some other variables, which are specified by low correlations concerning the measure of interest and the measure of other constructs. The correlation along with the mean and SD of the targeted variables is given in Table 2. The correlation results show a significant positive relationship between EI and academic performance (0.48, *p* < 0.01) and a significant negative relationship between EI and study stress (0.39, *p* < 0.05). Similarly, the correlation between EI and burnout (−0.35, *p* < 0.01). The relation between e-learning and academic performance (−0.36, *p* < 0.01), e-learning and study stress (−0.43, *p* < 0.01), and e-learning and burnout (−0.41, *p* < 0.001) shows a significant negative correlation, respectively.

**Table 2:** Correlations analysis.

**Variables**	**Mean**	**(SD)**	**α**	**1**	**2**	**3**	**4**	**5**
1. Academic performance	4.09	0.32	0.89	1				
2. Study stress	3.04	0.48	0.83	−0.17	1			
3. Burnout	3.02	0.35	0.87	−0.22^*^	0.27^*^	1		
4. EI	4.15	0.89	0.88	0.48^**^	−0.39^*^	−0.35^**^	1	
5. E-Learning	3.97	0.48	0.86	−0.36^**^	0.43^**^	0.41^***^	−0.23^**^	1

*EI, emotional intelligence*.

* *p < 0.05;*

** *p < 0.01;*

*** *p < 0.001*.

### Structural Model

The structure model measurement was run to examine the relationship between the targeted variables. The standardized regression analysis of the parameter paths is shown in Table 3. The analysis outcomes show a positive and significant influence of EI on academic performance (β = 0.37, *p* < 0.001). The EI shows a negative and significant impact on study stress (β = 0.18, *p* < 0.001) and burnout (β = 0.25, *p* < 0.01); therefore, our results supported Hypotheses 1, 2, and 3. Additionally, the effect of e-learning on academic performance (β = 0.21, *p* < 0.01) shows a positive significant relationships, supporting Hypothesis 4, the effects of e-learning on study stress (β = 0.45, *p* < 0.001) and burnout (β = 0.35, *p* < 0.05) shows a significantly negative relationship, consequently, our findings supporting Hypotheses 5 and 6, respectively.

**Table 3:** Statistical analysis results.

**Hypothesis**	**Beta**	***p*-value**	**Decision**
EI → Academic performance	0.37^***^	0.00	Accepted
EI → Study stress	−0.18^***^	0.00	Accepted
EI → Burnout	−0.25^**^	0.01	Accepted
E-Learning → Academic performance	−0.21^**^	0.01	Accepted
E-Learning → Study stress	0.41^***^	0.01	Accepted
E-Learning → Burnout	0.35^*^	0.05	Accepted
E-Learning ^*^ EI → Academic performance	0.21^*^	0.05	Accepted
E-Learning ^*^ EI → Study stress	−0.15^**^	0.01	Accepted
E-Learning ^*^ EI → Burnout	−0.17^**^	0.01	Accepted

*EI, emotional intelligence*.

* *p < 0.05;*

** *p < 0.01;*

*** *p < 0.001*.

### Moderation Analysis

Finally, the present study hypothesized the interaction (moderation) impact of EI with e-learning on academic performance, burnout, and study stress. The moderation analysis results show a significant impact of EI with e-learning on academic performance (β = 0.21; *p* < 0.05) as shown in Table 3; hence, hypothesis 7 is accepted.

Table 3 also provides a significant moderation impact of EI with e-learning on study stress (β = −0.15; *p* < 0.01) and burnout (β = −0.17; *p* < 0.01); therefore, our results supported Hypotheses 8 and 9, respectively. This signifies that EI with e-learning possesses a moderating impact on academic performance, study stress, and burnout.

In order to map the interaction, an average centered interaction term was shaped with the product of e-learning and EI and used for the interaction. When two standard variables are considered simultaneously, their interaction is a significant important predictor as provided in Table 3. Precisely, the interaction term anticipated academic performance (β = 0.21, Δ in *R*^2^ = 0.06, *p* < 0.05), study stress (β = −0.15, Δ in *R*^2^ = 0.04, *p* < 0.01), and burnout (β = −0.17, Δ in *R*^2^ = 0.05, *p* < 0.01) showing comprehensive support for the moderation impact in the hypothesized model. Specifically, EI moderated the relationship between e-learning and academic performance such that those who possess a high level of EI demonstrated a weaker negative connection between e-learning and academic performance. Contrarily, those individuals having low EI displayed a stronger negative relationship between e-learning and academic performance.

Our results found that EI shows a moderating impact between e-learning and study stress, indicating that students having less EI had a strong connection between e-learning and study stress, whereas students who possess high EI demonstrated a weak relationship between e-learning and study stress. Moreover, similar outcomes were found with burnout; e-learning and burnout show a stronger relationship with those students who have a low level of EI.

## Discussion

Currently, students observed that their academic performance had been interrupted due to the COVID-19 pandemic. This has forced thousands of students to stay away from their universities. The academic performance and psychological intentions of students are disturbed due to the lack of their college campuses, friends, professors, visiting the library, experimental activities in laboratories, group discussions, projects, etc.

Our finding confirms that Pakistani students show high psychological pressure toward online classes. They experience high levels of study stress and burnout and a low level of academic performance. In addition, students with high EI demonstrated a low level of study stress and burnout and better academic performance. The present study confirmed the influence of e-learning on academic performance, study stress, and burnout moderated by EI. Specifically, we discovered that students were suffering high psychological pressure from online classes facing low levels of study stress and burnout and high academic performance when they possess a high level of EI. Therefore, the present study supported all our hypotheses.

Furthermore, the results have identified two critical factors helping to understand the psychological intentions of Pakistani students during this COVID-19 pandemic: (a) the findings of this research add to the literature by signifying that e-learning and EI are critically important for the academic outcomes of a student. Students may become frustrated, show intent to give up or withdraw from taking online classes, and engage in negative behaviors toward their studies due to the psychological pressure of online classes; (b) the findings of this research advocate that EI can moderate e-learning, consequently, cause less study stress, burnout, and the consequence of enhancing academic performance. EI is a skill that can be developed through training and gradually increasing over the years. Yilmaz (2009) conducted a study. He argues that providing EI training programs to University students can help handle their psychological pressure and enhance the level of EI of students.

The results of the present study claim that those students who are better to handle and manage positive and negative sentiments are more capable of reacting accurately according to the existing requirements and effectively regulating emotions to increase their well-being due to experience psychological stress and burnout. The outcomes obtained in this study can confirm that it is important to include an emotional training program in the educational institutions and online classes to improve the learning process. Furthermore, the study of EI allows the student to take more potential possibilities to adjust to different uncertain situations and crises to obtain academic success (Herrera et al., 2020). Our results are parallel to the Lam and Kirby (2002) study; they argued that EI could assist students to resolve academic and daily problems more efficiently and managing psychological pressure effectively (Herrera et al., 2020; Moreno-Fernandez et al., 2020). Less EI individuals have been experiencing high psychological stress during the learning process (Adeyemo and Ogunyemi, 2005).

Cazan and Năstasă (2015) suggest that one of the students' major aspects is showing enthusiasm and building a strong connection with the academic curriculum. For successful academic achievements, students have to proficiently display skills to cope with stressful situations, keep a positive work attitude, and interact with professors. In addition, it should be understood that physical and emotional demands emphasize building interpersonal capabilities, but they also include productive relationships with friends, families, teachers, and other professionals in extremely hard and uncertain situations. Pedrosa et al. (2020) observed the connection between emotional challenges with the perspectives of students; they found that the psychological stress and burnout of students become more obvious. Consequently, regulation, controlling, and managing feelings and emotions are deemed compulsory for successful academic performance (Suleman et al., 2019; Halimi et al., 2020; MacCann et al., 2020).

## Conclusion and Limitations of the Study

To conclude, the present study examines the relationships among e-learning, EI, study stress, burnout, and academic performance of students, during the global pandemic. Our findings significantly support the previous literature that EI effectively copes with a negative effect and stress arising from the COVID-19 pandemic (Chandra, 2020). Similarly, the results of Estrada et al. (2021) also parallel with our findings that EI enhances academic performance and reduces psychological stress among students. Based on the empirical results, the authors suggest that EI development and training programs might be included in the academic curriculum for students, which may help to control the serious effects (study stress and burnout) link to high levels of psychological pressure experienced by students during online classes. EI training and development programs can enhance the ability of a student to control and mitigate stressful situations (Drigas and Papoutsi, 2020), and also support the students to improve their social adjustment and academic performance (Wang, 2019). As a result, this might increase the ability of students to face and manage uncertain situations effectively and, ultimately, will enhance their academic performance.

This study had some limitations. First, rely on self-report assessment in this research could raise concerns of response biases. Second, to advance the generalizability of the current conclusions, a future study can imitate this model on other professionals, such as University teachers, organization leaders, and employees of multinational companies. Moreover, this research investigated the moderating role of EI between e-learning and the psychological intentions of overseas students. Therefore, it would be fascinating to observe the impact of other variables (e.g., IQ and cultural intelligence). A noticeable limitation of the present study includes the sample size. We gathered and analyzed data from only 10 Chinese University students stranded in Pakistan, and the sample size was 387 students, which is not big data. Further study may consider a large sample of data, collecting from several different countries.

## Data Availability Statement

The raw data supporting the conclusions of this article will be made available by the authors, without undue reservation.

## Ethics Statement

Ethical review and approval was not required for the study on human participants in accordance with the local legislation and institutional requirements. Written informed consent for participation was not required for this study in accordance with the national legislation and the institutional requirements.

## Author Contributions

All the authors contributed to the conceptualization, formal analysis, investigation, methodology, writing of the original draft, and writing review and editing. All the other authors contributed to the formal analysis, investigation, methodology, and writing review and editing. All authors have read and agreed to the published version of the manuscript.

## Conflict of Interest

The authors declare that the research was conducted in the absence of any commercial or financial relationships that could be construed as a potential conflict of interest.

## Publisher's Note

All claims expressed in this article are solely those of the authors and do not necessarily represent those of their affiliated organizations, or those of the publisher, the editors and the reviewers. Any product that may be evaluated in this article, or claim that may be made by its manufacturer, is not guaranteed or endorsed by the publisher.
